# Biosynthesis of silver nanoparticles employing *Trichoderma harzianum* with enzymatic stimulation for the control of *Sclerotinia sclerotiorum*

**DOI:** 10.1038/s41598-019-50871-0

**Published:** 2019-10-04

**Authors:** Mariana Guilger-Casagrande, Tais Germano-Costa, Tatiane Pasquoto-Stigliani, Leonardo Fernandes Fraceto, Renata de Lima

**Affiliations:** 1 Laboratory for Evaluation of the Bioactivity and Toxicology of Nanomaterials, University of Sorocaba (UNISO), Sorocaba, São Paulo Brazil; 2 Laboratory of Environmental Nanotechnology, State University of São Paulo (UNESP), Sorocaba, São Paulo Brazil

**Keywords:** Assay systems, Nanoparticles

## Abstract

Biogenic synthesis of silver nanoparticles employing fungi offers advantages, including the formation of a capping from fungal biomolecules, which provides stability and can contribute to biological activity. In this work, silver nanoparticles were synthesized using *Trichoderma harzianum* cultivated with (AgNP-TS) and without enzymatic stimulation (AgNP-T) by the cell wall of *Sclerotinia sclerotiorum*. The nanoparticles were evaluated for the control of *S*. *sclerotiorum*. The specific activity of the *T*. *harzianum* hydrolytic enzymes were determined in the filtrates and nanoparticles. Cytotoxicity and genotoxicity were also evaluated. Both the nanoparticles exhibited inhibitory activity towards *S*. *sclerotiorum*, with no new sclerotia development, however AgNP-TS was more effective against mycelial growth. Both the filtrates and the nanoparticles showed specific enzymatic activity. Low levels of cytotoxicity and genotoxicity were observed. This study opens perspectives for further exploration of fungal biogenic nanoparticles, indicating their use for the control of *S*. *sclerotiorum* and other agricultural pests.

## Introduction

The biogenic synthesis of silver nanoparticles is an attractive nanotechnological alternative to other chemical and physical methods, offering simplicity, relatively low cost, lower generation of toxic waste, lower energy consumption, and higher yields. Bacteria, yeasts, fungi, plant extracts, and algae can be used in this type of synthesis, acting as reducing agents and stabilizers^1^. The ability of such organisms to alter the chemical nature of metals is due to their development of mechanisms of defense against toxic agents, with the products being nanoparticles with lower toxicity^2^. The process of extracellular biogenic synthesis is probably mediated by the nitrate reductase enzyme, which acts in the reduction of metals, leading to the formation of nanoparticles^3^.

Fungi have been extensively used in the synthesis of metallic nanoparticles due to their ease of handling and cultivation, high biomass production, and the secretion of large quantities of metabolites, enzymes, and extracellular proteins. These biomolecules not only act in the reduction process of the metal precursor, but also form a capping on the nanoparticles, hence providing better control of size and stability^4–6^. The biogenic synthesis of silver nanoparticles can be achieved using a wide range of filamentous fungi and yeasts, including *Fusarium oxysporum*^7^, *Aspergillus* sp^8^., *Trichoderma harzianum*^9,10^, *Trichoderma asperellum*^11^, *Beauveria bassiana*^12,13^, *Penicillium* sp^14^. and *Candida albicans*^15^. Depending on the species of fungus used in the synthesis (due to the different extracellular enzyme profiles), the nanoparticles produced can have different physicochemical characteristics and biological activities^16^, making them promising materials for use in the areas of health, agriculture, and the environment.

In addition to the different characteristics of nanoparticles according to the fungal species used, differences can also occur using the same species under different culture conditions. Variations of culture medium composition, pH, temperature, growth time, and agitation can alter the metabolism of the microorganisms, consequently influencing the physicochemical characteristics of the nanoparticles and the composition of the capping, which can either enhance the synthesis or make it unviable^17,18^.

*Trichoderma harzianum* is a mycoparasitic filamentous fungus used as a biological control agent against phytopathogens that affect the production of several agriculturally important plant species. Its main mechanism of action is by the growth of hyphae and the release of hydrolytic enzymes that degrade the cell wall of the target fungus. These enzymes include chitinases, N-acetyl-β-D-glucosaminidases, and proteases, which are fundamental in the mycoparasitism process^19–21^. The secretion of these enzymes is enhanced in the presence of phytopathogenic fungi, so it is possible to induce enzyme production by mimicking mycoparasitism, cultivating *Trichoderma* spp. in culture media supplemented with cell wall material from the phytopathogen^22,23^. Due to the favorable characteristics of *T*. *harzianum* for the control of phytopathogens, together with its easy handling, this fungus has been extensively explored in the fields of biotechnology and nanotechnology, opening avenues for the development of new products and applications^24^. Fungi of the genus *Trichoderma* spp. present the NADH co-enzyme and NADH-dependent enzymes such as nitrate reductase, which is important in the synthesis of both the nanoparticles and the cappings that confer greater stability^3,25^.

In previous work, silver nanoparticles synthesized using the filtrate of *T*. *harzianum* presented inhibitory activity against the phytopathogenic fungus *Sclerotinia sclerotiorum*, which causes white mold disease^10^. In the present work, two types of silver nanoparticles were synthesized, considering that the characteristics of biogenic nanoparticles can differ according to the composition of the culture medium in which the reducing and stabilization agent is cultivated. The first type was obtained using the filtrate of *T*. *harzianum* with enzyme production stimulated by the cell wall of *S*. *sclerotiorum*, while the other type was obtained using the filtrate from *T*. *harzianum* grown in the absence of the cell wall.

*S*. *sclerotiorum* affects the production of crops including soybean, tomato, lettuce, beans, and sunflower, among others, causing agriculture economical losses in several countries around the world, including all the continents^26,27^. Resistant structures (sclerotia) can remain viable in the soil for decades and can be widely distributed, leading to plant diseases that cause annual economic losses of around 1.47 billion dollars in Brazil^28^. In the United States, the disease caused losses of about 2.8 million tons of soybean between 2010 and 2014, the equivalent to 1.2 billion dollars, with a devastating epidemic in 2009^29,30^. Yield losses in different crops are also observed in Canada, India and several european countries^27^. Considering the severity of the impacts of *S*. *sclerotiorum*, evaluation was made of the *in vitro* activities of the AgNP-TS and AgNP-T nanoparticles against this phytopathogen, as well as their possible effects on *T*. *harzianum*. Determinations were also made of the physicochemical characteristics of the formulations, the specific activities of the *T*. *harzianum* hydrolytic enzymes, and possible toxic effects of the nanoparticles.

## Materials and Methods

### Biogenic synthesis of the nanoparticles using *Trichoderma harzianum*

The cultivation of *T*. *harzianum* was performed using commercial Ecotrich^TM^ wettable powder (1 × 10^10^ CFU/g, Ballagro) in potato-dextrose agar medium, in the absence of light, at ambient temperature. Disks of the mycelium were transferred to potato-dextrose broth and potato-dextrose broth supplemented with 0.5% cell wall of the *S*. *sclerotiorum* phytopathogen as a source of carbon and nitrogen^21,23^. The cultures were kept under agitation at 150 rpm for 12 days, after which the biomasses were weighed, transferred to ultrapure water, and kept under the conditions described above for 72 h. The samples were then vacuum-filtered and AgNO_3_ was added to a final concentration of 1 mM^10^. Two different types of nanoparticle were obtained, with one synthesized using the filtrate from *T*. *harzianum* stimulated with *S*. *sclerotiorum* (AgNP-TS) and the other synthesized using the filtrate from *T*. *harzianum* without stimulation (AgNP-T). Employing this methodology it is possible to obtain dispersions of 2,000 mL of nanoparticles.

### Physicochemical characterization of the nanoparticles

After the synthesis, the nanoparticles and the corresponding *T*. *harzianum* filtrates were analyzed by UV-visible spectroscopy in the wavelength range between 200 and 800 nm, with resolution of 1 nm, using a Shimadzu Multispec 1501 spectrophotometer. The pH values of the nanoparticles and the filtrates were measured using a pH meter (HMMPB-210).

The hydrodynamic diameters and polydispersities of the nanoparticles were measured by dynamic light scattering (DLS) and the zeta potentials were determined by microelectrophoresis, using a ZS90 particle analyzer (Malvern Instruments). The analyses were performed in triplicate, at 25 °C, with a fixed angle of 90°. The sizes and concentrations (NPs.mL^−1^) of the nanoparticles were also determined by nanoparticle tracking analysis (NTA), using a NanoSight LM10 cell (Malvern Analytical) coupled to a camera and controlled with NanoSight v. 2.3 software. The nanoparticles were diluted 50-fold in ultrapure water and 5 measurements were performed for each sample.

### Hydrolytic enzyme specific activity assays

The specific activities of the *T*. *harzianum* hydrolytic enzymes β-1,3-glucanase, N-acetylglucosaminidase (NAGase), chitinase, and acid protease were determined using microplate assays in which the enzyme sources were the *T*. *harzianum* filtrates obtained with and without stimulation, as well as the corresponding nanoparticles (AgNP-TS and AgNP-T). The protein concentrations were first determined using the method of Bradford^31^, with bovine serum albumin (0.125, 0.250, 0.500, and 1.000 mg.mL^−1^) as standard. The assays of the four enzymes employed the methodology described by Qualhato *et al*.^21^.

### Biological activities of the nanoparticles towards *Sclerotinia sclerotiorum* and effects on *Trichoderma harzianum*

For evaluation of the ability of the nanoparticles to control the mycelial growth and sclerotia development of *S*. *sclerotiorum*, Petri dishes were prepared with potato-dextrose agar culture medium containing AgNP-TS and AgNP-T at final concentrations of 3 × 10^9^ NPs.mL^−1^, in triplicate. Controls were prepared using potato-dextrose agar alone and inoculated with *T*. *harzianum* (127 μg.mL^−1^). After solidification of the agar, a viable sclerotium was placed in the center of each plate, followed by keeping at room temperature for 15 days, with a photoperiod of 12 h. At the end of the period, the mycelium growth halo was measured and the number of new sclerotia was counted.

Evaluation was also made of possible effects of the nanoparticles on *T*. *harzianum*, given the importance of ensuring the viability of this biological control agent for both the control of phytopathogens and the induction of plant growth and development. For this, plates were prepared with culture media containing the nanoparticles, as described above, which were inoculated with *T*. *harzianum* at 127 μg.mL^−1^, in duplicate. The cultures were kept at ambient temperature for 15 days, in the dark, followed by growth analysis.

### Viability/cytotoxicity and genotoxicity assays using the nanoparticles

The cytotoxic effects of the nanoparticles were evaluated using the V79 (chinese hamster lung fibroblast), 3T3 (albino Swiss mouse embryo fibroblast), and HaCat (human keratinocyte) cell lines. The techniques employed were the tetrazolium reduction assay (MTT test for mitochondrial activity), image cytometry (cell viability, apoptosis, and necrosis), and the trypan blue exclusion test (cell viability). For the MTT assay, the cells were previously cultured in Dulbecco’s modified eagle medium (DMEM), plated in 96-well plates (5 × 10^4^ cells per well), and exposed for 24 h to the nanoparticles at concentrations from 0.1 × 10^9^ to 3.5 × 10^9^ NPs.mL^−1^. The wells were than washed with phosphate buffer saline (PBS), MTT solution (5 mg.mL^−1^) was added, and the plates were left for 3 h. Finally, the samples were fixed with dimethylsulphoxide (DMSO) and were read using a microplate reader at 540 nm. Image cytometry analyses of cell viability, apoptosis, and necrosis were performed using an apoptosis kit with Annexin V Alexa Fluor^TM^ 488 and propidium iodide (Invitrogen). The cells were exposed for 1 h to the nanoparticles at a concentration of 3 × 10^9^ NPs.mL^−1^ and were then prepared as specified by the manufacturer. The readings were obtained using a Tali^TM^ image cytometer (Invitrogen). For the trypan blue exclusion assay, the cells were exposed for 1 h to the nanoparticles at 3 × 10^9^ NPs.mL^−1^, followed by staining with trypan blue and counting the cells using an optical microscope, considering cells stained blue to be dead. The positive controls were cells exposed to 0.5 M hydrogen peroxide, while the negative controls were cells kept in culture medium without any exposure.

The genotoxic potentials of the nanoparticles were determined using the *Allium cepa* assay, as described by Lima *et al*.^32^. The roots were exposed for 24 h to AgNP-TS and AgNP-T at concentrations of 1 × 10^10^ and 3 × 10^9^ NPs.mL^−1^, followed by fixing in ethanol:acetic acid (3:1). For preparation of the slides (in triplicate), the roots were hydrolyzed in 1 M HCl, at 60 °C, and stained with Schiff’s reagent. The meristematic regions were cut, stained with acetic carmine, and crushed under cover slips. The cells were observed under an optical microscope, with counting of the cells in division and those that presented chromosomal alterations, hence obtaining the mitotic index (MI) and chromosomal alteration index (AI) values.

The comet assays to evaluate the effects of AgNP-TS and AgNP-T were performed using an adaptation of the methodology described by Singh *et al*.^33^. The previously cultured cells were exposed for 1 h to the nanoparticles at concentrations of 3 × 10^9^ NP.mL^−1^, followed by mixing with agarose, application to pre-gelatinized slides, and keeping for 1 h in lysis solution. Following neutralization, the slides were kept for 20 min in electrophoresis buffer and were subsequently submitted to electrophoresis for 20 min at 22 V and 300 mA. The slides were then fixed, stained with silver, and visual scoring was performed under an optical microscope.

### Statistical analyses

Statistical treatment of the data employed one-way analysis of variance (ANOVA) followed by Tukey’s test, with a significance level of p < 0.05. These analyses were performed with GraphPad Prism 7.0 software.

## Results and Discussion

### Physicochemical characterization of the nanoparticles

**Figure 1:**
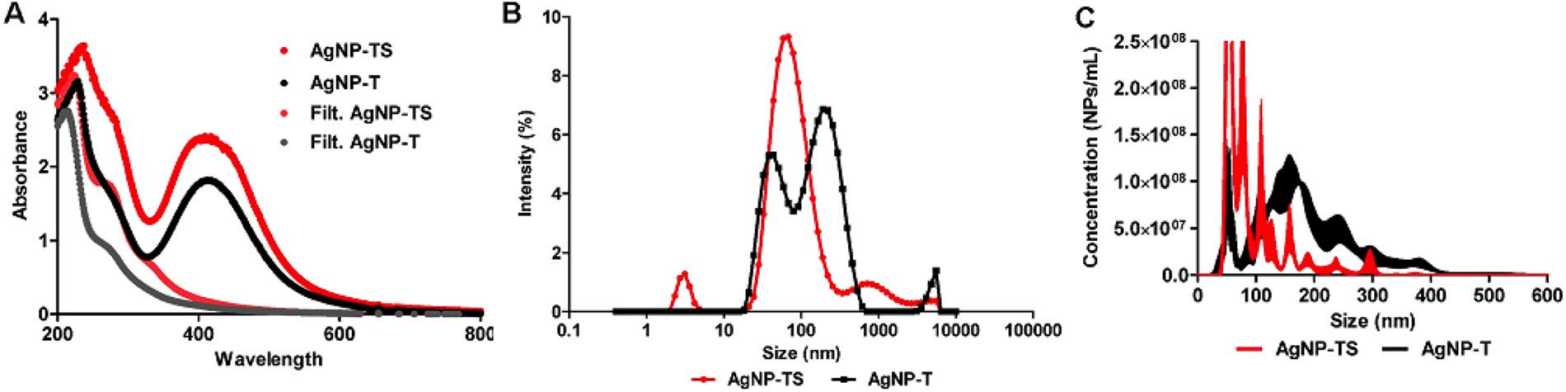
Physicochemical characterization of the AgNP-TS and AgNP-T nanoparticles. (**A**) UV-vis spectra of the nanoparticles and the corresponding filtrates, (**B**) Hydrodynamic diameters obtained by the DLS technique, (**C**) Nanoparticle sizes and concentrations obtained by the NTA method.

Silver nanoparticles were successfully synthesized using the filtrates from *T*. *harzianum* cultivated in the presence and absence of the cell wall of *S*. *sclerotiorum*, which resulted in the nanoparticles AgNP-TS and AgNP-T. The filtrates showed color change from light yellow to reddish-brown 72 h after addition of AgNO_3_, due to the surface plasmon resonance of the silver^34^. The synthesis was confirmed by UV-visible spectroscopy, with peaks obtained at 409 and 413 nm for AgNP-TS and AgNP-T, respectively (Fig. 1A), indicating the presence of elemental silver^35,36^. Analysis of the filtrates used in the synthesis revealed peaks at 221 and 215 nm for the samples obtained with and without stimulation by the cell wall of *S*. *sclerotiorum*, respectively. These absorbance peaks observed for the filtrates were also present in the spectra for the nanoparticles, reflecting the presence of filtrate proteins in their compositions. In previous studies, Phanjom and Ahmed^37^ reported peaks at 210 and 260 nm for filtrate of the fungus *Aspergillus oryzae* used for the synthesis of silver nanoparticles, which were attributed to amides and amino acid residues, respectively. Durán *et al*.^38^ used *Fusarium oxysporum* to synthesize silver nanoparticles, with peaks at 265 nm attributed to aromatic amino acids of proteins released into the filtrate, which contributed to silver nitrate reduction and stabilization of the nanoparticles. Ballotin *et al*.^39^ found two main UV-vis bands for silver nanoparticles synthesized using *Aspergillus tubingensis*, with one at 440 nm, confirming formation of the silver nanoparticles, and another at 280 nm, attributed to aromatic amino acids such as tryptophan, tyrosine, and phenylalanine residues, which composed the protein capping of the nanoparticles. The presence of peaks corresponding to proteins and amino acid residues in both the filtrates and the capped nanoparticles confirmed the release of these compounds from the fungal biomass dispersed in water, indicating that they were involved in the process of reduction of the metal ions, leading to the formation and stabilization of the nanoparticles.

The hydrodynamic diameters, polydispersity indices, and zeta potentials of the nanoparticles were determined by the DLS and microelectrophoresis techniques. The AgNP-TS and AgNP-T nanoparticles presented mean hydrodynamic diameters of 57.02 ± 1.75 and 81.84 ± 0.67 nm, respectively (Fig. 1B). The polydispersity and zeta potential values were 0.49 ± 0.01 and −18.70 ± 3.01 mV for AgNP-TS and 0.52 ± 0.00 and −18.30 ± 1.73 mV for AgNP-T, respectively. The difference in size between the nanoparticles synthesized using the filtrates of *T*. *harzianum* with and without stimulation by the *S*. *sclerotiorum* cell wall could have been due to the different culture medium compositions. Various factors have been reported to influence nanoparticle physicochemical characteristics, including the composition of the medium in which the reducing agent is cultivated^40^.

The NTA technique was also used to determine the sizes and concentrations of the nanoparticles (Fig. 1C), resulting in different concentrations for AgNP-TS and AgNP-T, with particle sizes of 88.0 ± 7.3 and 182.5 ± 6.9 nm, respectively. After characterization, the stock concentration of the nanoparticles was standardized at 1 × 10^10^ NPs.mL^−1^, in order to facilitate the activity and toxicity evaluations. Durán *et al*.^38^ reported the presence of a capping around biogenic nanoparticles synthesized using the fungus *Fusarium oxysporum*, which avoided aggregation. In agreement with the previous report, the nanoparticles synthesized in the present work by the biogenic method presented a capping of proteins and other biomolecules derived from the organism used as the reducing agent (*T*. *harzianum*). This capping provided stability to the nanoparticles, so that they maintained their original size and did not aggregate^35^.

The pH of the medium in which the synthesis occurs (in this case, the filtrates) can affect the size and morphology of the nanoparticles. A higher pH leads to smaller nanoparticles with spherical morphology, while a lower pH results in larger nanoparticles in the forms of rods and prisms^41^. In the present case, both filtrates presented pH of 7.2, while no significant changes were observed after the synthesis, with near-neutral values of 7.2 and 7.3 for AgNP-TS and AgNP-T, respectively. It has been reported that for non-biogenic nanoparticles, the pH and size of newly synthesized nanoparticles influence their reactivity, with lower values of these parameters indicating greater possibility of dissolution and ionization, which could lead to increased toxic effects^42^.

### Determination of hydrolytic enzyme specific activity

**Figure 2:**
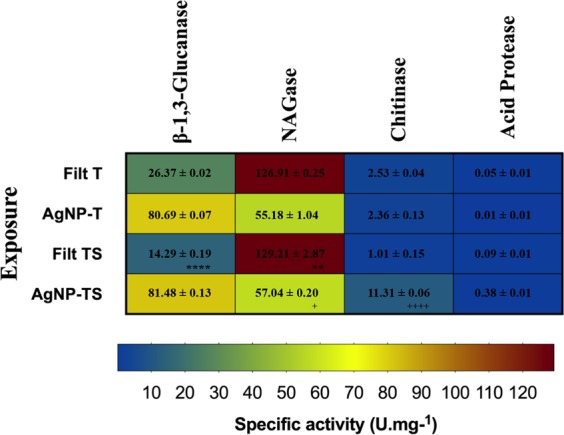
Specific activities (U/mg) of the hydrolytic enzymes of *Trichoderma harzianum* present in the nanoparticles and the corresponding filtrates. Statistically significant difference (p < 0.05) is indicated by * for comparison of Filt T (filtrate without stimulation) and Filt TS (filtrate with stimulation) and + for comparison of AgNP-T and AgNP-TS. More symbols indicate a higher statistical significance.

The assays used to determine the specific activities of the *T*. *harzianum* hydrolytic enzymes β-1,3-glucanase, NAGase, chitinase, and acid protease detected the activities of these enzymes in both the filtrates, as well as in the AgNP-TS and AgNP-T nanoparticles (Fig. 2). It was not possible to compare the activity levels of the filtrates and the corresponding nanoparticles, due to the concentration changes in the synthesis processes. However, it could be seen that the highest specific activity was obtained for NAGase, followed by β-1,3-glucanase for both the filtrates and the nanoparticles. The specific activity of chitinase and acid protease were also observed with lower values, especially for acid protease.

Small differences were observed between the enzymatic activities for the filtrates obtained with and without stimulation by the cell wall of *S*. *sclerotiorum*, and between the activities for different nanoparticles. Higher specific activity of the NAGase and chitinase was observed for AgNP-TS in comparison with AgNP-T. Regarding to the filtrates, the exposure of *T*. *harzianum* to the cell wall of *S*. *sclerotiorum* stimulated the specific activity of the enzyme NAGase.

The effectivity of the biogenic nanoparticles for the control of *S*. *sclerotiorum* can be attributed to the specific activity of the hydrolytic enzymes present in the capping of the nanoparticles. In a previous study, Guilger *et al*.^10^ evaluated the effects of commercial AgNPs, non-biogenic and uncapped, and these nanoparticles were not able to control fungal development.

Geraldine *et al*.^23^ evaluated the specific activity of the hydrolytic enzymes NAGase, acid phosphatase, β-glucosidase, lipase, β-1,3-glucanase and proteases in the filtrates of different *Trichoderma* species and strains after exposure to *S*. *sclerotiorum* cell wall. All the species and strains showed enzymatic activity in different proportions and in a varied way, with the highest activities for NAGase, acid phosphatase, and protease.

Qualhato *et al*.^21^ also reported the production and secretion of β-1,3-glucanase, NAGase, chitinase, acid phosphatase, acid proteases and alginate lyase in the filtrates of *Trichoderma* spp. grown in the presence of the cell wall of the pathogenic fungi *Fusarium solani*, *Rhizoctonia solani* and *S*. *sclerotiorum*. To our knowledge, no prior studies have examined the specific activity of fungal hydrolytic enzymes in samples of biogenic silver nanoparticles.

### Biological activity of the nanoparticles against *Sclerotinia sclerotiorum* and effects on *Trichoderma harzianum*

**Figure 3:**
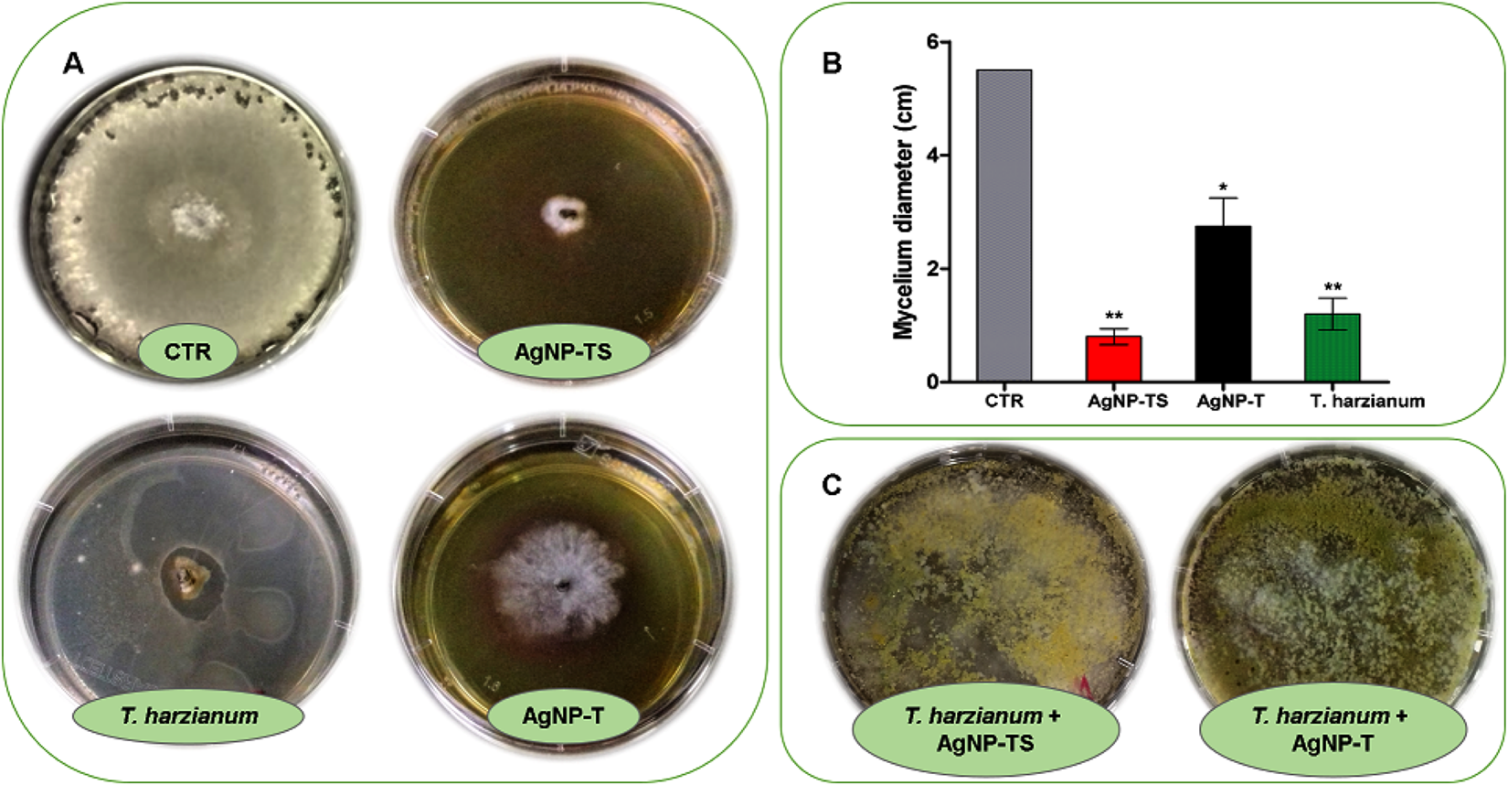
Biological activities of the AgNP-TS and AgNP-T nanoparticles (3 × 10^9^ NPs.mL^−1^) towards *S*. *sclerotiorum* and *T*. *harzianum*. (**A**) Control of mycelium growth and sclerotia, (**B**) Mycelium diameter, (**C**) Effect on *T*. *harzianum*. Statistically significant difference (p < 0.05) is indicated by * for comparison of the exposures to the control (CTR), with **indicating a higher statistical significance.

The biological activity results showed that the AgNP-TS and AgNP-T nanoparticles presented potential for the control of *S*. *sclerotiorum*, since mycelial growth was reduced and there was no formation of new sclerotia. Treatment using *T*. *harzianum* also resulted in inhibition of *S*. *sclerotiorum*. As shown in Fig. 3A, many new sclerotia were formed from the precursor sclerotium at the edges of the control plate (CTR), with a mean of 116.5 ± 7.7 sclerotia, while the AgNP-TS and AgNP-T plates showed no new sclerotia and reduced mycelial growth. The greatest mycelial inhibition was provided by AgNP-TS, which capping was derived from the filtrate obtained using stimulation to enhance the production of *T*. *harzianum* hydrolytic enzymes (Fig. 3B).

The superior control of mycelial growth achieved using AgNP-TS could have been related to the smaller nanoparticle size, given that previous work has found that a smaller nanoparticle size is associated with higher activity^43^. An additional consideration is that these nanoparticles were synthesized using filtrate from a culture in which there had been stimulation of the production of hydrolytic enzymes that could act in degradation of the cell walls of the phytopathogen. The better control of mycelial growth by AgNP-TS can be attributed to the higher activity of the enzymes NAGase and chitinase from these nanoparticles.

The inhibition of microorganisms by silver nanoparticles is due to the strong surface oxidative activity of the particles and the release of ions^44^. However, in the case of biogenic nanoparticles, there may also be a contribution of the stabilizing capping derived from the reducing organism, which deserves further investigation.

Previous studies have investigated the use of silver nanoparticles for controlling agricultural phytopathogens including *S*. *sclerotiorum*, with effective inhibitory activity observed *in vitro*^45–48^. However, the mechanism of action of nanoparticles is not yet understood^49^. Krishnaraj *et al*.^46^ tested different concentrations of silver nanoparticles against the phytopathogenic fungi *Alternaria alternata*, *Sclerotinia sclerotiorum*, *Macrophomina phaseolina*, *Rhizoctonia solani*, *Botrytis cinerea*, and *Curvularia lunata*, with inhibitory effects observed against all these species. Despite such promising results, there have been no previous studies concerning the biogenic synthesis of nanoparticles with stimulation of the metabolism of the biological agent employed for the reduction and stabilization. This is important, since enzyme production by fungi is directly influenced by the conditions under which the organisms are cultivated^50^.

*Trichoderma harzianum* is widely used as a biological control agent therefore it is important to evaluate possible effects of nanomaterials against this fungus^51^. In our study the nanoparticles caused no alteration of *T*. *harzianum* growth (Fig. 3C), which was an important finding, indicating the possibility of its use in combination with the nanoparticles.

The use of nanotechnological products for the control of pests in agriculture is considered to be safer than employing traditional agrochemicals, since it helps to avoid excessive accumulation in the environment of chemicals that can cause residual toxicity^49^. An additional consideration is that the use of chemical fungicides can lead to the development of resistance by phytopathogens^52,53^. Nonetheless, it is possible that the release of new nanomaterials into the environment could become a problem for humans and other organisms^54^. For this reason, it is vital that their potential toxicities towards biological systems should be thoroughly investigated, in order to ensure the safe development of effective new nanomaterials^55^. The nanometric size of nanoparticles enables their internalization within the cells of living organisms, which might lead to serious consequences^56^.

### Viability/cytotoxicity and genotoxicity evaluation of the nanoparticles

**Figure 4:**
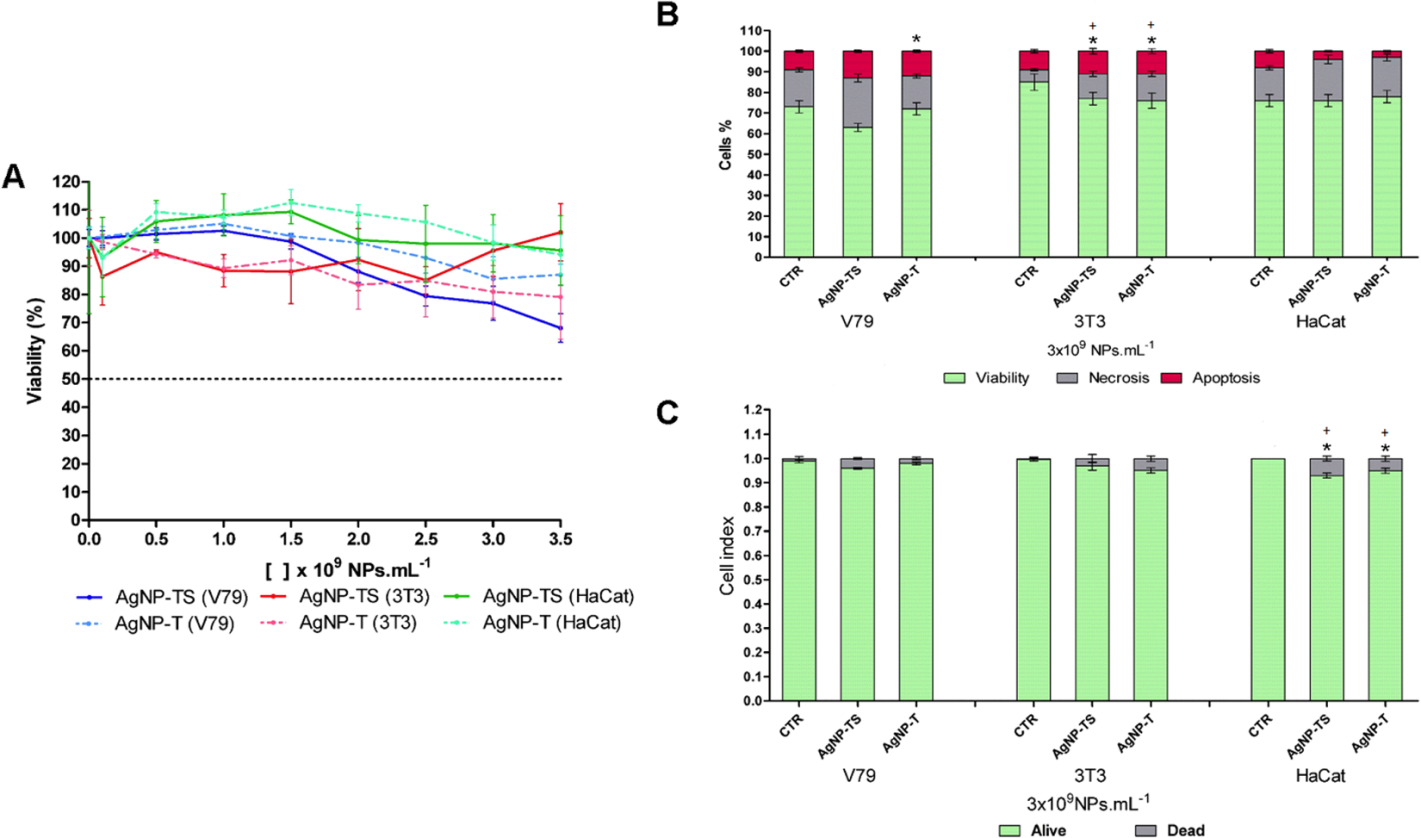
Cytotoxicity evaluation of the AgNP-TS and AgNP-T nanoparticles. (**A**) Tetrazolium reduction test (MTT), (**B**) Image cytometry determination of cell viability, necrosis, and apoptosis with statistically significant difference (p < 0.05) indicated by * for comparison of viability to the control (CTR) and + for comparison of apoptosis to the control (CTR), (**C**) Trypan blue exclusion test with statistically significant difference (p < 0.05) indicated by * for comparison of alive cells to the control (CTR) and + for comparison of dead cells to the control (CTR).

Evaluation of the cytotoxic effects of the nanoparticles using the MTT test, which indicates the mitochondrial activity of the cells, revealed differences in the viability percentages for the different cell lines. However, despite presenting different sensitivities, no IC50 values were observed for any of the samples, indicating low cytotoxicity of the nanoparticles in the range of exposure concentrations employed (Fig. 4A), including the concentration of interest for the control of *S*. *sclerotiorum* (3 × 10^9^ NPs.mL^−1^). These results were consistent with the direct analyses of cytotoxicity using the image cytometry (Fig. 4B) and trypan blue (Fig. 4C) methods, although it is important to take the different exposure periods into consideration (24 h for the MTT test and 1 h for the image cytometry and trypan blue methods). In the image cytometry tests, the two types of nanoparticle caused similar levels of apoptosis and necrosis, with the HaCat cell line presenting viability equivalent to that of the control. The results of the trypan blue exclusion assays indicated that exposure to both types of nanoparticle led to significant decreases of the viabilities of the 3T3 and HaCat cells, while the V79 cells only showed decreased viability when exposed to AgNP-TS. In comparison of the two types of nanoparticle, AgNP-TS caused the lowest viabilities of the V79 and HaCat cells.

Silver nanoparticles can alter normal cellular functions, affect membrane integrity, and initiate programmed death processes^57^. This cytotoxicity may arise from physicochemical interactions between silver atoms and the functional groups of cellular proteins^58^. However, although these effects apply to silver nanoparticles in general, the nanoparticles obtained using biogenic synthesis present specific characteristics related to the organism used in the reduction process, with the cytotoxicity generally being lower than observed for commercial nanoparticles and solutions containing silver ions^59^. Skladanowski *et al*.^60^, using the MTT test, reported an absence of cytotoxicity in L929 mouse fibroblasts exposed to silver nanoparticles synthesized using *Streptomyces* sp. NH28.

**Figure 5:**
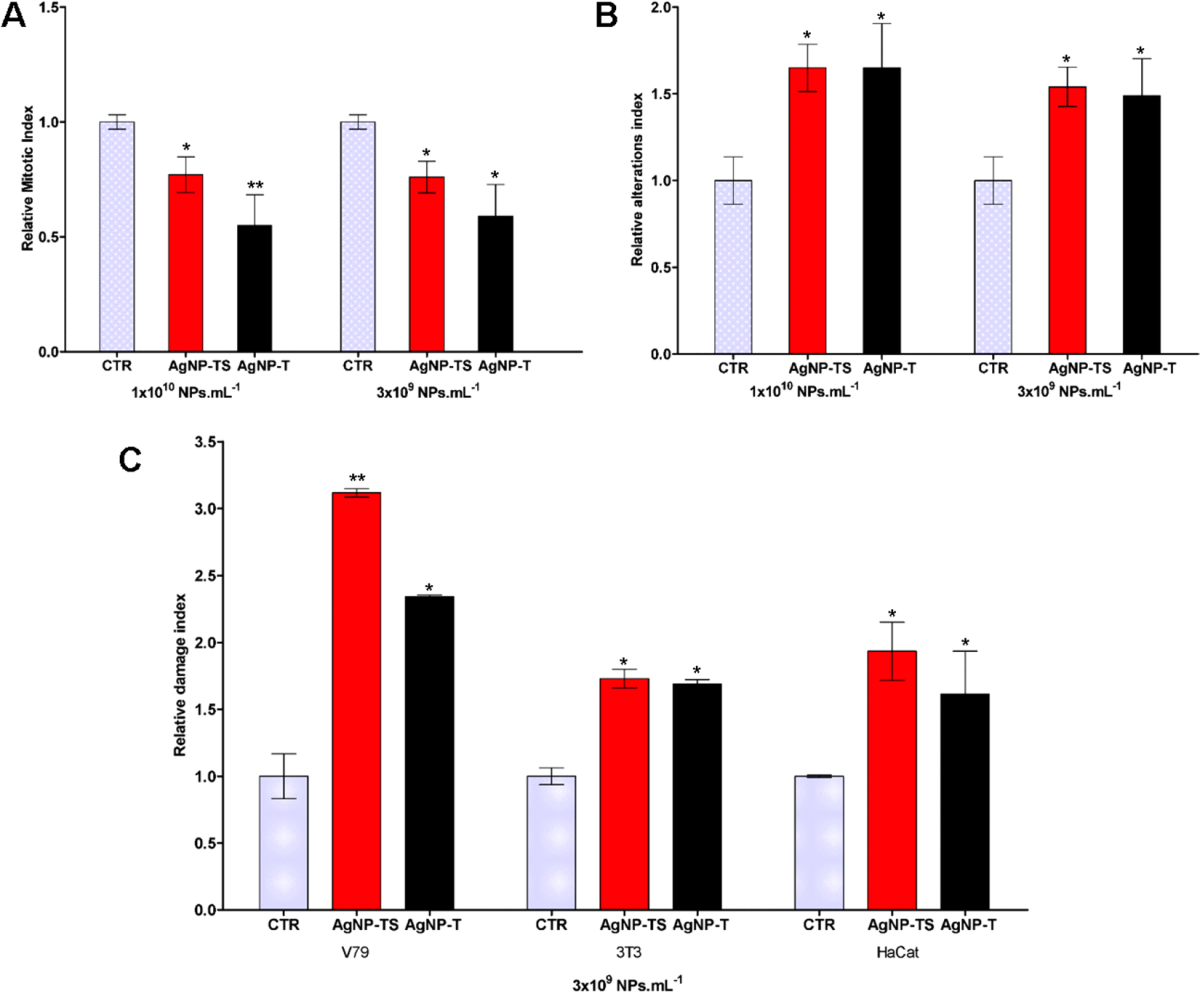
Evaluation of the cytotoxicity and genotoxicity of AgNP-TS and AgNP-T. (**A**) Relative mitotic index using the *Allium cepa* assay, (**B**) Relative chromosomal alteration index using the *Allium cepa* assay, (**C**) Relative DNA damage index using the comet assay. Statistically significant difference (p < 0.05) is indicated by * for comparison of the exposures to the control (CTR), with **indicating a higher statistical significance.

The *Allium cepa* assay was used to investigate possible genotoxicity of the nanoparticles and mitotic index alteration. At the two exposure concentrations tested, both types of nanoparticle caused the mitotic index values to decrease, compared to the control (Fig. 5A). The alteration indices indicated that both AgNP-TS and AgNP-T caused increased chromosomal alterations, at the exposure concentrations, with no difference between the effects of the two nanoparticle types (Fig. 5B).

The results of the *Allium cepa* assay were consistent with those obtained in a similar trial using the plant species *Drimia polyantha*, where decreases of the mitotic index and the presence of chromosomal alterations were found following exposure to the highest concentrations of biogenic silver nanoparticles synthesized using the plant *Getonia floribunda*^61^. Similar results were obtained using the *Allium cepa* assay to evaluate the effects of low concentrations of silver nanoparticles synthesized using the plant *Swertia chirata*^62^. The toxic effects caused by silver nanoparticles may be due to the generation of reactive oxygen species and DNA damage, leading to apoptosis, as well as the release of Ag^+^ ions, which depends on the rate of dissolution of the nanoparticles within the cells^63–65^.

Further evaluation of the genotoxicity of the nanoparticles was performed using comet assays to determine the extent of DNA damage in the V79, 3T3, and HaCat cell lines. Both types of nanoparticle caused increases of the damage indices for the three cell lines, compared to the control, with the V79 cells showing the greatest sensitivity to AgNP-TS (Fig. 5C).

## Conclusions

Silver nanoparticles were successfully synthesized using the filtrates from *T*. *harzianum* cultivated in the presence and absence of the cell wall of *S*. *sclerotiorum*, which resulted in nanoparticles with different physicochemical characteristics. Both AgNP-TS and AgNP-T were able to control the growth of *S*. *sclerotiorum*, with inhibition of mycelial growth and prevention of the formation of new sclerotia. The most effective control of mycelial growth was achieved using AgNP-TS, which could be attributed to the smaller hydrodynamic diameter of the nanoparticles, as well as a possible effect of the biomolecules from the capping of the nanoparticles, which were derived from the filtrate obtained using stimulation of the enzymatic production of *T*. *harzianum*. The effect of the capping could have acted in synergy with the effect of the nanoparticles themselves. Both types of nanoparticle presented low levels of cytotoxicity and genotoxicity towards V79, 3T3, and HaCat cell lines.

This study provides new perspectives for the development of techniques involving the biogenic synthesis of metallic nanoparticles, considering the biological activity potentials of not only the metal (in this case, silver), but also the biomolecules and organic compounds derived from the organisms used in the synthesis, which compose the capping on the nanoparticles. The promising results obtained for the control of *S*. *sclerotiorum* highlight this system among the emerging applications of nanotechnology in the agricultural sector.
